# An Order Insertion Scheduling Model of Logistics Service Supply Chain Considering Capacity and Time Factors

**DOI:** 10.1155/2014/530678

**Published:** 2014-09-02

**Authors:** Weihua Liu, Yi Yang, Shuqing Wang, Yang Liu

**Affiliations:** College of Management and Economics, Tianjin University, Tianjin 300072, China

## Abstract

Order insertion often occurs in the scheduling process of logistics service supply chain (LSSC), which disturbs normal time scheduling especially in the environment of mass customization logistics service. This study analyses order similarity coefficient and order insertion operation process and then establishes an order insertion scheduling model of LSSC with service capacity and time factors considered. This model aims to minimize the average unit volume operation cost of logistics service integrator and maximize the average satisfaction degree of functional logistics service providers. In order to verify the viability and effectiveness of our model, a specific example is numerically analyzed. Some interesting conclusions are obtained. First, along with the increase of completion time delay coefficient permitted by customers, the possible inserting order volume first increases and then trends to be stable. Second, supply chain performance reaches the best when the volume of inserting order is equal to the surplus volume of the normal operation capacity in mass service process. Third, the larger the normal operation capacity in mass service process is, the bigger the possible inserting order's volume will be. Moreover, compared to increasing the completion time delay coefficient, improving the normal operation capacity of mass service process is more useful.

## 1. Introduction 

Faced with presently growing demand for customized logistics services, many logistics enterprises expand their business beyond mass service and change logistics service mode to provide customized service. Specifically, these enterprises attempt to provide mass customization logistics services (MCLS) instead of mass logistics services [1]. In order to meet customized service requirements and achieve necessary mass service capabilities in the MCLS environment, logistics enterprises usually organize unions and integration [2]. And the competitiveness of the LSSC depends on the ability to offer mass customization service with the cost as low as possible through reasonable scheduling [3].

In LSSC scheduling, time scheduling is quite important, and it should balance customer demand and logistics service capacity. Compared with production supply chain, service cannot be reserved or buffered in the form of tangible products. Therefore, operation of service supply chain is much more easily influenced by outside environment, especially when there is an order insertion. Order insertion refers to the situation where new orders arrive and are required to be inserted into a scheduled order sequence when production capacity is fixed and resources are limited, which is common in the practice of service industry [4]. The insertion of new jobs into an existent schedule, as well as most of the other types of disruptions, may require the total or partial rescheduling of previously allocated and new jobs. For example, as one of the biggest express companies in China, Yuantong Express Company always faces the problem of insertion scheduling. Normally, at twelve o'clock at noon every day, Yuantong Express will collect all the packages in the morning and forward them to customers at transit centre together. However, some emergent orders happen occasionally, so Yuantong Express will consider the factors of time and operation cost, judge whether it could carry out insertion scheduling, and then make a new scheduling planning. Due to the abruptness and urgency of order insertion, it will be more difficult to make time schedule in LSSC considering logistics service capacity limitation, time requirements, and increased cost. Thus, how to arrange service capacity and operation time reasonably becomes a realistic problem every LSI faces.

While order insertion has been studied by many scholars in production supply chain scheduling so far, it is still a relatively new issue in service supply chain. Though some scholars have become interested in service supply chain scheduling problem recently, for example, [3, 5, 6], they consider more normal scheduling situation than order insertion. Obviously, time scheduling with order insertion is much more complex and thus worthwhile to research.

Based on the literature review and specific practical observation about logistics enterprises, it is found that under MCLS environment, LSI needs to focus on solving three problems during time scheduling within order insertion, which are the lack of existed research and thus the focus of paper.

First, due to the abruptness and urgency of order insertion, LSI will change the original order schedule for a rescheduled one. Therefore, it is necessary to discuss whether it is feasible to insert new orders with full consideration of original time schedule.

Second, if the new order to be inserted is similar to original orders, then how could the LSI make the best use of this similarity and reschedule the time under the MCLS environment. Factors such as logistics service capacity, time requirement from customers, and operation cost caused by order insertion have to be dealt with properly in the model.

Third, it is of great significance for the LSI managers to figure out what factors that do have influence on order-inserting decisions and what are the specific influence rules in practical scheduling process. With the help of these rules, LSI could deal with order insertion problem better.

These problems mentioned above would be answered in this paper. Based on the research of Liu et al. [3] and Liu et al. [6], this paper has further discussed order similarity coefficient and the order insertion process in LSSC, which contributes to two essential constraints. Furthermore, with full consideration of both capacity and time two factors, an order insertion scheduling model of LSSC has been established, aiming to minimize the average unit volume operation cost of the LSI and maximize the average satisfaction degree of FLSPs. All constraints in our model are different from those in previous researches. Under these conditions, some interesting findings are obtained. First, whether the new order could be inserted or not depends on its volume that will further affect supply chain comprehensive performance. In particular, supply chain will get the best performance when the inserted order's volume is equal to the surplus of the normal operation capacity of mass service process. Besides, time requirement from customers will also influence supply chain comprehensive performance, and some allowable delay in completion time appropriately will contribute to better performance. What is more, compared to increasing completion time delay coefficient, improving normal operation capacity of mass service process is more useful in increasing the upper limit of possible inserting order volume. Therefore, choosing to increase normal operation capacity of mass service process is a prior strategy when LSI needs to solve new order insertion problem.

The rest of the paper is organized as follows.  Section 2 systematically reviews the existing researches of order insertion in supply chain scheduling. In Section 3, the problem and basic assumptions are described in detail and notations used in model building are listed specifically.  Section 4 gives an order insertion scheduling model of LSSC considering capacity and time factors. In Section 5, the model solution is calculated within genetic algorithm. In Section 6, numerical examples are given to explore the influence of parameters related to new order on the time scheduling performance.  Section 7 is a concluding section.

## 2. Literature Review

Our research is mainly concerned about the order insertion scheduling of LSSC under the environment of MCLC. Thus, the literature review is mainly related to MC and order insertion scheduling. Our research aims will be proposed after summarizing the literature development and its deficiencies.

### 2.1. Researches on MC and Scheduling in LSSC

Since Pine [7] proposed that mass customization mode would become the new frontier in business competition in 1993, MC mode has increasingly become the mainstream mode of operation after nearly 20 years of development and application. Due to its significant improvement on operational performance, mass customization has been extensively studied and applied in the field of production supply chain. So many scholars conducted monographic studies. Fogliatto et al. [8] reviewed the literature on MC production in detail since the 1980s. From the view of the current domestic and international research progress, researches on MC were mainly developed within the MC production mode in manufacturing industry, including MC mode and its product development; see, for example, [9], production planning and control technology in MC; see, for example, [10], cost study of MC; see, for example, [11], research on the factors and conditions that influence MC; see, for example, [12].

The studies on the supply chain scheduling with the mass customization production mode was a new upsurge in recent years. Operation scheduling under MC environment is more dynamic and of more complexity. Most of the researches on supply chain scheduling have been focusing on the manufacturing industry and have achieved further results. In 2003, Hall and Potts [13] published a paper named “Supply chain scheduling: batching and delivery,” which is an earlier systematical research on the supply chain scheduling model. Many earlier studies on supply chain scheduling pay attention to the job shop scheduling within a single enterprise, for example, [14]. And they are mainly concerned about the arrangement of processing procedures and order operation sequence. Some scholars also are concerned about the coordination of assembly system in manufacturing enterprise; see, for example, [15]. However, the studies on the supply chain scheduling with the mass customization production mode was a new upsurge in recent years; see, for example, [16].

Many scholars have carried out targeted researches on the supply chain scheduling. Cost is the primary factor considered in many above researches; see, for example, [17]. And most of these researches assume that the order completion time required by customers or the delivery time required by suppliers was fixed. But as an important index reflecting supply chain agility, customers' time requirements might change in a lot of cases [18, 19] or the operation time requirements to LSPs are not with strict limitation but allow a certain amount of variation; see, for example, [20]. Thus, it is necessary to consider the influence of service completing time ahead of schedule or delay caused by customers or LSPs on the scheduling results [21]. Besides cost objective, punctual delivery of service order and FLSP's satisfaction also have a direct influence on customer satisfaction. Therefore, it is necessary to consider the influence of the different importance degree of different objective functions on the supply chain performance. However, the current literature has not addressed this issue.

Although now the research on supply chain scheduling under MC environment becomes more and more complete, the one on service supply chain field is still significantly deficient. Similar to the manufacturing supply chain, researches on service supply chain are mainly focusing on the service process scheduling; see, for example, [22] and the order assignment scheduling; see, for example, [2]. The most related researches to this paper are Liu et al. [3] and Liu et al. [6], in which time scheduling problem in LSSC is discussed. But they only focused on the scheduling of a decided set of orders without taking order insertion situation and the influence of capacity support on time scheduling result into consideration. Thus, in general, research on time scheduling is still far from sufficient. It is necessary to study the time scheduling problem in service supply chain field (especially in LSSC field).

### 2.2. Researches on Order Insertion Scheduling of Supply Chain

Order insertion is a special and important content in supply chain scheduling research. In production supply chain field, order insertion problem has gained much attention. Order insertion refers to inserting a new arrival order into a scheduled order sequence on the premise that the production capacity has been allocated. Sometimes the new inserted order will replace original ones and form a new order sequence. Therefore, previous order insertion scheduling researches mainly focused on two research emphasis. One is the order insertion method. Since order insertion process may break original production schedule, it may cause other original orders to be delayed. Thus, it is necessary and useful to explore reasonable order insertion methods. At present, common method of inserting a new order includes “right shift,” “insertion in the end,” and “total rescheduling.” Some researches combined order insertion problem with other disturbance factors such as machine breakdown and boiled down. Another focus is the problem to decide the priority of inserting order.

Compared to that in make-to-stock production mode, order insertion problem in make-to-order mode has gained some attention as well. To find out the influencing factors of order insertion decision is very important to build order insertion models. Some scholars explored these influential factors under different situations, such as time, cost, scheduling efficiency, and scheduling stability. For example, in order insertion model, time constraint is often regarded as an important considering factor and decreasing time delay is always regarded as a crucial scheduling goal. Duron et al. [23] used operation time and lead time to characterize different original orders and assumed that new order insertion operation may cause delay in original orders' delivery. Duron et al. [24] tried to reduce original order delay caused by new order insertion operation through a real-time approach. Besides, many scholars regarded minimizing supply chain cost as a frequently used objective in order insertion model; see, for example, [25]. Gomes et al. [26] studied order insertion problem in make-to-order industries. They took scheduling efficiency and stability index as measures of the influence of rescheduling process on original schedule and introduced a reactive scheduling algorithm to update scheduling table.

As can be seen from the above review of the literatures, the existing researches have three deficiencies. First, in production supply chain, research on order insertion is mainly focused on the priority algorithm of inserting orders, which aim at finding excellent algorithm to improve optimizing efficiency. Moreover, many of the literatures assume that supply chain capacity can afford the new order insertion requirement and other original orders' satisfaction degree is not affected, but real situations are not the same. Second, new inserting order has its own features both in structure and required operations. The existing researches do not consider the factors that whether the new inserting order can be operated together with original orders considering these feathers. Meanwhile, it is not be discussed whether the FLSP's capacity can afford inserting operation. Third, in the existing researches on MC service supply chain, order insertion scheduling research considering time factors is rare. Thus, based on these three deficiencies, this paper will fully consider the similarity between original orders and the new inserting one as well as the influence of service capacity of supply chain on order insertion decision. In the MC service environment, this paper will deeply explore decision problem that whether a new arrival order can be inserted into original orders to be rescheduled. Furthermore, some useful references are offered for better study on order insertion issue.

## 3. Problem Description and Model Assumptions

In this section, the problem and basic assumptions are described in detail. Notations used in model building are listed as well. In Section 3.1, both the problems involved in the model and the decision process of order insertion are described. In Section 3.2, important assumptions in our model are listed specifically. In Section 3.3, related notions defined in this paper are provided in detail as well as the scheduling logic in our model.

### 3.1. Problem Description

In a two-echelon LSSC with one LSI and many FLSPs, LSI accepts customers' service orders and hands them to multiple FLSPs to operate. And LSI faces multiple customer service orders at the same time and each logistics service order consists of multiple service processes, which could be divided into two types, that is, personalized service process and large-scale service process, where whether to integrate the large-scale service process of customer *i* and customer *j*  (*j* ≠ *i*) to be operated together or not can be chosen. These two kinds of service processes are called “mass service process” and “customized service process,” respectively, in this paper.

Since customer orders arrive in sequence, after the scheduling process of an original set of order has been finished, new order may occur to be inserted into schedule, including urgent order and order which asked to be operated first by customers. At this time, LSI needs to first decide whether this new arrival order could be inserted while synthetically considering the characteristic of new orders and FLSPs' capacity. Furthermore, scheduling decision model and method of order insertion problem should be thought over by LSI.

**Figure 1:**
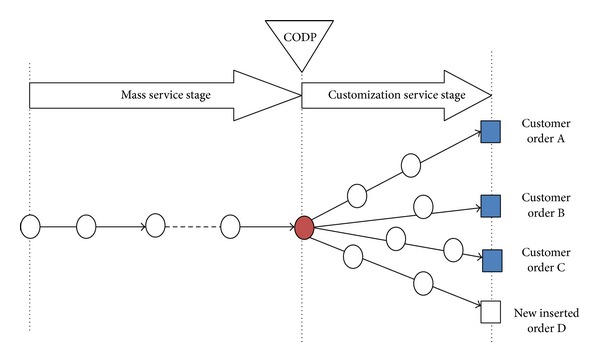
Customer orders' operation processes schematic diagram of general LSSC.

**Figure 2:**
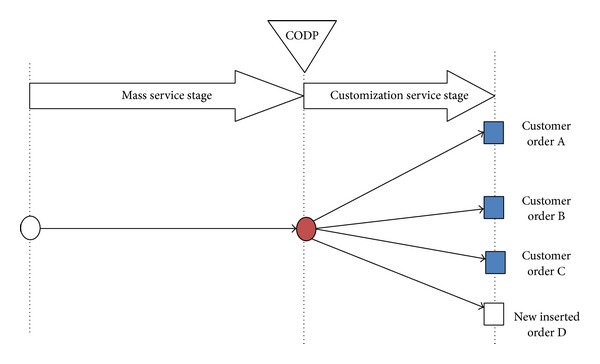
Customer orders' operation processes schematic diagram of LSSC which is simplified.

First, a specific example is used to illustrate this scheduling problem. See Figure 1; there are three original customer orders (order A, order B, and order C) whose partial service processes can be operated together in mass mode due to the similarity in their service content. Service processes after CODP will be operated in customized mode, respectively. Upon arrival of new customer order D, LSI needs to decide whether to insert this new order based on synthetically consideration of this new order's and original orders' characteristic as well as the FLSPs' operation capacity. For the convenience of study, it is necessary to simplify the process as shown in Figure 2. It is assumed that mass process is operated by FLSP 1 and the customized process of the *j*th customer order is operated by the *j*th FLSP of customized stage, respectively. The FLSP 1 completes the mass process and it has capacity limit. Namely, in a normal completion time *T*_1_, FLSP 1 can finish an order whose volume is $\bar{N}$. If the FLSP is required to operate a task whose volume is more than $\bar{N}$, then order setup time increases or capacity reorganization is needed to be carried out. We assume that there is no capacity limit for the *j*th FLSP (in this example *j* = 2,3, 4,5) because of customization service.

One thing needs to be noted. Mass customization service could be normally divided into two stages: mass service stage and customization service stage. For the mass service stage, multiple orders are integrated and operated together, so it is necessary to consider the factors of time, operation cost, and service process for all the multiple orders. For the customization service stage, each order is finished by customization process; there is no relationship among multiple orders. Obviously, order insertion scheduling is an activity that new orders are required to be inserted into a scheduled order sequence. Thus insertion scheduling always be carried out in mass service stage but not happens in customization service stage.

**Figure 3:**
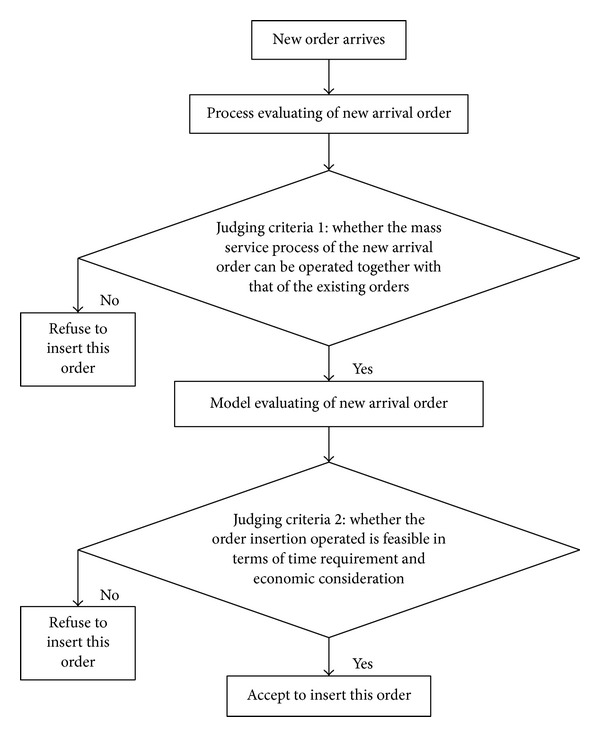
Determining process of new order's insertion decision.

Because the new inserted order is unpredictable, whether it can be inserted into original schedule should be considered. Therefore, the judging criteria are proposed in the Figure 3.

Judging criterion 1 is as follows: whether the mass service process of the new arrival order can be operated together with that of the existing orders; specifically, whether similarity between the mass process of new arrival order and the original ones exists. If the answer is positive, then turn to judging criteria two.

Judging criterion 2 is as follows: whether the order insertion operated is feasible in terms of time requirement and economic consideration, namely, use the time scheduling model proposed in this paper to carry through model judgment. If this model has solution, then the order insertion decision is feasible by the model judgment and FLSPs can carry on order insertion operation according to scheduling results. If this model has no solution, then this new order cannot be inserted into the original schedule. For example, it may not meet time requirement or profit requirement.

In this paper, two judging criteria are proposed in order to judge whether a new order could be inserted into the original schedule or not. If it is impossible to insert new order, a completely new scheduling plan should be put out.

The model parameters and variables are summarized in Table 1.

### 3.2. Model Assumptions

In order to build our model conveniently, some important assumptions are proposed as follows.

**Assumption 1 .**

Customer orders arrive at different time. Original orders arrive first and the new inserted order arrives later. Before arrival of new order, original orders have been scheduled. FLSPs have set their normal operation time and necessary capacity plan for each process according to schedule table. The new arrived order needs to go through two judging criteria mentioned above, but in this paper, we only focus on the second judging criterion and assume that the new order have passed the first judging criteria. That means it is assumed that the new inserted order could be scheduled with original orders together. If new arrival order is inserted into the original ones to be operated together, the normal operation time of original orders may be compressed or delayed.

**Assumption 2 .**

In our model, we assume that there is only one new arrived order that needs to be inserted and do not consider multiorder insertion problem. If the new order is able to be inserted, then in the rescheduling process, we view all the orders to have the same priority, since all the orders are operated together but not operated one by one in the mass process.

**Assumption 3 .**

If order is delayed, LSIs will be punished by customer; while if the order is finished in advance, they will not. Within the endurable time of customer, the unit time punish cost is *C*_*j*_^delay^. If the actual completion time is *T*_*j*_, then the punish cost is *C*_*j*_^delay^[*T*_*j*_−*T*_*j*_^exp⁡^,0]^+^. If the time delay is beyond customer's durable time, then customer order cannot continue being operated and supply chain collapses.

**Assumption 4 .**

Each provider can compress or delay their operation time through increasing input capacity, such as increasing vehicle or lengthening working time in order to meet customer's time requirement. Correspondingly, LSI needs to pay extra cost for capacity input increase. Extra cost for unit time compression or delay in mass process is *C*_1_^ext^ and that in customized process is *C*_2*j*_^ext^.

**Assumption 5 .**

In mass service process, the case may occur that service capacity is insufficient due to FLSP's capacity limitation. But in customized service process, since each service order is operated by a specialized provider, service capacity is assumed to be always sufficient.

**Assumption 6 .**

Influence of new inserted order on CODP is not considered in this paper; that is, the CODP is assumed unchanged.

### 3.3. Preparation for Model Building

#### 3.3.1. Order Similarity Coefficient

Similarity between original orders and new inserted order must be taken into consideration when dealing with order insertion problem. In this paper, *λ* is used todenote order similarity coefficient. Analysis on order similarity is a crucial step to consider order insertion decision. In production supply chain, clustering analysis on different orders is often carried out according to product's modular construction. But in service supply chain, different service orders have many differences and it is hard to choose a modular measure index like tangible products. Therefore, this paper will focus on the analysis of service order similarity coefficient.

Take the research findings of order similarity of tangible product for reference; see [27–30]; and taking service product features into consideration, a service order similarity coefficient is defined as a product of three indexes, which are customer demand similarity *λ*_1_ (namely, time requirement similarity coefficient), service procedure similarity coefficient *λ*_2_ (such as service standard similarity and service process similarity), and customer service product similarity coefficient *λ*_3_ (such as function similarity and structure similarity of service product). The detailed calculation method for each kind of similarity coefficient will be introduced as follows.

(*1) Customer Demand Similarity Coefficient λ*_1_. In the supply chain time scheduling, time requirement is the most important customer requirement. In this paper, time requirements similarities of different orders are used to denote customer demand similarity coefficient. The smaller the completion time requirement gap between original orders and new inserted order is, the more similar they are. And the average completion time of all the original orders is regarded as another benchmark. The closer the completion time requirement of new inserted order is, the bigger the similarity is. Detailed calculation method is shown in

(1) $$\begin{array}{c} \lambda_{1}=\{\begin{array}{cc} \frac{\left(1/J_{0}\right)\sum_{j=1}^{J_{0}}T_{j}^{\exp}}{T_{J_{0}+1}^{\exp}}, & \text{when}\;\frac{1}{J_{0}}\overset{J_{0}}{\underset{j=1}{\sum }}T_{j}^{\exp}\leq T_{J_{0}+1}^{\exp}\\ \frac{T_{J_{0}+1}^{\exp}}{\left(1/J_{0}\right)\sum_{j=1}^{J_{0}}T_{j}^{\exp}}, & \text{when}\;\frac{1}{J_{0}}\overset{J_{0}}{\underset{j=1}{\sum }}T_{j}^{\exp}>T_{J_{0}+1}^{\exp}. \end{array} \end{array}$$

(*2) Service Procedure Similarity Coefficient λ*_2_. There are many differences for service procedure of different orders. Service procedure similarity between original orders and new inserted order has significant influence on the feasibility of order-inserting operation when facing order insertion decision. Generally speaking, similarity of service procedure consists of three parts. First is service standard similarity, such as service quality standard and standard for service staff. Second is service stage similarity, for example, whether there are some similar service stages between original orders and new inserted order. The third is service process similarity. For example, if original orders have load or unload process but the new inserted order does not, then they are relatively different and the service step similarity coefficient is relatively small.

(*3) Customer Service Product Similarity λ*_3_. It mainly refers to function and structure similarity of service product. For example, if operation for original orders and new inserted order are both transportation services for household chemicals, then they are of much similarity due to belonging to the same category. If original orders are transportation for steel and new inserted order is for cotton, obviously, they have less similarity.

Note that, since service procedure similarity coefficient *λ*_2_ and customer service product similarity *λ*_3_ are both difficult to be quantized. Therefore, values of *λ*_2_ and *λ*_3_ can be obtained by questionnaire or based on LSI's experience. Their value ranges from 0 to 1. Take the previous researches for [29, 30], the similarity coefficient is denoted as *λ* = *λ*_1_ *λ*_2_ *λ*_3_. Thus, the order similarity coefficient can be shown as

(2) λ=λ1λ2λ3={(1/J0)∑j=1J0Tjexp⁡TJ0+1exp⁡λ2λ3,when  1J0∑j=1J0Tjexp⁡≤TJ0+1exp⁡TJ0+1exp⁡(1/J0)∑j=1J0Tjexp⁡λ2λ3,when  1J0∑j=1J0Tjexp⁡>TJ0+1exp⁡.

#### 3.3.2. Preparation Time for New Order

Different volume of new inserted order will make different influence on supply chain scheduling result. Obviously, the more the volume is, the more the operation stress of supply chain system will be. Along with the increase of new inserted order's volume, resource that needed to be prepared will increase. For example, FLSPs need to prepare more transportation vehicles or warehouses. Therefore, besides the increasing cost, new inserted order will cause increase of preparation time to redeploy resource. The influence of new inserted order's volume on time scheduling result should be reflected in this model. In general, increased order preparation time is positively correlated with three factors. The first one is the normal operation time of original orders in mass service process *T*_1_. Second one is extra order volume ${\left[\sum_{j=1}^{J_{0}+1}N_{j}-\bar{N}\right]}^{+}$. Last one is the order similarity coefficient *λ*. *t* is used to denote the increased order preparation time caused by new inserted order as shown in

(3) t=[∑j=1J0+1Nj−N¯]+N¯(1−λ)T1={[∑j=1J0+1Nj−N¯]+N¯(1−(1/J0)∑j=1J0Tjexp⁡TJ0+1exp⁡λ2λ3)T1,           when  1J0∑j=1J0Tjexp⁡≤TJ0+1exp⁡[∑j=1J0+1Nj−N¯]+N¯(1−TJ0+1exp⁡(1/J0)∑j=1J0Tjexp⁡λ2λ3)T1,           when  1J0∑j=1J0Tjexp⁡>TJ0+1exp⁡,

where ${\left[\sum_{j=1}^{J_{0}+1}N_{j}-\bar{N}\right]}^{+}=\max\left(\sum_{j=1}^{J_{0}+1}N_{j}-\bar{N},0\right)$. If ${\left[\sum_{j=1}^{J_{0}+1}N_{j}-\bar{N}\right]}^{+}<0$, then it is unnecessary to prepare for extra logistics service resource, such as vehicle. On the contrary, if ${\left[\sum_{j=1}^{J_{0}+1}N_{j}-\bar{N}\right]}^{+}>0$, it means that the new inserted order's volume is more than surplus of supply chain capacity; then extra preparation of logistics service resource is necessary and order preparation time will increase.

#### 3.3.3. Order Rescheduling and Operation Time Logic

New inserted order will cause rescheduling of LSSC on the premise that the original order has been scheduled. Since the insertion of new order may cause completion time delay of original orders, it becomes a focusing goal for LSI to try to meet customer orders' time requirement through possible operation time compression. It is necessary to not only ensure original orders to be operated according to customer requirement but also guarantee profit increased after inserting a new order.

Based on the analysis above, the real completion time after order insertion could be decided by calculation. Namely, real completion time of the *j*th order is *T*_*j*_ = order preparation time (directly influenced by inserted order) + order operation time (which is able to be compressed or delayed); *j* = 1,2,…, *J*_0_ + 1. Note that preparation time cannot be compressed, while operation time is compressible. As reflected in our model, this compressible (or deferrable) extra operation time is our scheduling content. Therefore, the decision variables are FLSP's extra operation time which aim at meeting customers' time requirement after inserting a new order, that is, *T*_*i*_^ext^  (*i* = 1,2).

## 4. Model Building 

This section will establish an order insertion scheduling model of LSSC considering capacity and time factors under order insertion situation.  Section 4.1 will describe main model objectives, which are to minimize LSI's unit operation cost and to maximize the average satisfaction degree of all the providers after order insertion.  Section 4.2 will present the main model constraints, which are time constraint, FLSP's satisfaction degree constraint, and capacity limit constraint.

### 4.1. Optimization Objectives of the Scheduling Model

#### 4.1.1. Objective 1: To Minimize LSI's Unit Operation Cost after Order Insertion

The objective to minimize LSI's unit operation cost after order insertion could be expressed as

(4) $$\begin{array}{c} \mathrm{Min}\;Z_{1}=\frac{\left(f_{1}+f_{2}+f_{3}\right)}{\sum_{j=1}^{J_{0}+1}N_{j}}, \end{array}$$

where *f*_1_ is the total cost of normal operation in mass process and customization process. Consider

(5) $$\begin{array}{c} f_{1}=C_{1}T_{1}\overset{J_{0}+1}{\underset{j=1}{\sum }}N_{j}+\overset{J_{0}+1}{\underset{j=1}{\sum }}\left(C_{2j}T_{2j}N_{j}\right). \end{array}$$

*f*_2_ is the extra operation cost in mass process and customized process. Consider

(6) $$\begin{array}{c} f_{2}=C_{1}^{\text{ext}}\left|T_{1}^{\text{ext}}\right|\overset{J_{0}+1}{\underset{j=1}{\sum }}N_{j}+\overset{J_{0}+1}{\underset{j=1}{\sum }}\left(C_{2j}^{\text{ext}}\left|T_{2j}^{\text{ext}}\right|N_{j}\right). \end{array}$$

*f*_3_ is punishment cost for order completion time delay. Consider

(7) $$\begin{array}{c} f_{3}=\overset{J_{0}+1}{\underset{j=1}{\sum }}\left[C_{j}^{\text{delay}}{\left(T_{j}-T_{j}^{\exp}\right)}^{+}\right],\\ T_{j}=T_{1}+T_{1}^{\text{ext}}+T_{2j}+T_{2j}^{\text{ext}}+\frac{{\left[\sum_{j=1}^{J_{0}+1}N_{j}-\bar{N}\right]}^{+}}{\bar{N}}\left(1-\lambda \right)T_{1}, \end{array}$$

where [*f*(*x*)]^+^ = max⁡{0, *f*(*x*)}, the same below. *T*_*j*_ is actual completion time of the *j*th customer order which consists of three parts, that is, completion time of mass process *T*_1_ + *T*_1_^ext^, completion time of customized process *T*_2*j*_ + *T*_2*j*_^ext^, and increased order preparation time caused by new order insertion $\left({\left[\sum_{j=1}^{J_{0}+1}N_{j}-\bar{N}\right]}^{+}/\bar{N}\right)\left(1-\lambda \right)T_{1}$.

#### 4.1.2. Objective 2: To Maximize the Average Satisfaction Degree of All the Providers

FLSP's satisfaction degree is quite hard to be quantized in reality, but it is very important in scheduling. Here, two aspects are chosen to measure FLSP's satisfaction degree, which are the product of quantity satisfaction degree of service capacity and service time satisfaction degree [3].

(*1) FLSP's Satisfaction Degree of Mass Process.* (1) Quantity satisfaction degree of service capacity *S*_quantity,1_ reflects FLSP's utilization status in terms of service quantity in mass service process. When order volume is less than the upper service capacity limit, the bigger the utilization of service capacity is, the more satisfied the provider is. But when order volume exceeds the upper limit of service capacity, satisfaction degree will decrease because of the overload operation status. According to Assumption 4 and Liu et al. [2], FLSP's satisfaction degree of mass process *S*_quantity,1_ can be presented as follows:

(8) Squantity,1={Squantity,10+1N¯∑j=1J0+1Nj(1−Squantity,10),     when  0<∑j=1J0+1Nj≤N¯N¯∑j=1J0+1Nj, when  ∑j=1J0+1Nj>N¯,

where *S*_quantity,1_^0^ means the initial satisfaction degree of provider in mass process when order volume is more than 0. It differs with different providers. $\bar{N}$ is the upper limit of FLSP's normal capacity in mass process.

(2) Service time satisfaction degree *S*_time,1_ reflects the satisfaction degree of provider for the service time schedule made by LSI. Generally speaking, when providers are operating as the schedule appointed in advance, their satisfaction degree is the highest. If LSI asks them to compress or delay their completion time suddenly, indeed, providers will become less satisfied. Therefore, the degree of closeness between actual completion time and normal operation time is used to denote FLSP's service time satisfaction degree. Consider

(9) $$\begin{array}{c} S_{\text{time},1}=\{\begin{array}{cc} \frac{T_{1}}{T_{1}+T_{1}^{\text{ext}}}, & T_{1}^{\text{ext}}\geq 0\\ \frac{T_{1}+T_{1}^{\text{ext}}}{T_{1}}, & T_{1}^{\text{ext}}<0. \end{array} \end{array}$$

Thus, FLSP's satisfaction degree of mass process is shown as *S*_1_ = *S*_time,1_ × *S*_quantity,1_.

(*2) FLSP's Satisfaction Degree of Customized Process.* According to Assumption 4, for customized process, operation volume of original orders is not affected by new order insertion. Thus, it is unnecessary to redeploy capacity. The *j*th FLSP's satisfaction degree is only related to service time factor. Consider

(10) S2j=Stime,2j{T2jT2j+T2jext,when  T2jext≥0T2j+T2jextT2j,when  T2jext<0,          j=1,2,…,J0+1.

With the satisfaction time of mass and customized process integrated, the average satisfaction degree for all providers could be calculated as

(11) $$\begin{array}{c} \mathrm{Max}Z_{2}=\frac{S_{1}+\sum_{j=1}^{J_{0}+1}S_{2j}}{1+J_{0}+1}=\frac{S_{\text{time},1}S_{\text{quantity},1}+\sum_{j=1}^{J_{0}+1}S_{2j}}{1+J_{0}+1}. \end{array}$$

### 4.2. Constraints of the Scheduling Model

#### 4.2.1. Constraint 1: To Meet Customers' Time Requirement

It is required that each customer order's completion time cannot be longer than the upper limit *T*_*j*_^exp⁡^(1 + *β*_*j*_) set by the corresponding customer. Consider

(12) Tj=T1+T1ext+T2j+T2jext+[∑j=1J0+1Nj−N¯]+N¯λT1≤Tjexp⁡(1+βj),

where *β*_*j*_ indicates the delay coefficient of the order completion time permitted by the *j*th customer for its order.

#### 4.2.2. Constraint 2: LSI's Increased Profit Resulted by New Order Insertion Is Larger than 0

This constraint shows the necessary condition that LSI is willing to carry out order insertion decision. In other words, the price paid by customer for its inserted order must exceed order insertion cost of LSI. Then constraint 2 can be presented as follows:

(13) Δpro=Nj+1F−[(f1+f2+f3) −(C1T1∑j=1J0Nj+∑j=1J0(C2jT2jNj))]>0,

where (*f*_1_ + *f*_2_ + *f*_3_) stands for the total cost of all the orders after new order inserted into original ones.

#### 4.2.3. Constraint 3: Each FLSP's Satisfaction Degree Is Larger than Its Lower Limit

Consider

(14) $$\begin{array}{c} S_{1}=S_{\text{time},1}S_{\text{quantity},1}\geq S_{1}^{L},\\ S_{2j}=S_{\text{time},2j}\geq S_{2j}^{L}. \end{array}$$

#### 4.2.4. Constraint 4: The Upper Limit of Capacity in Mass Process

According to Assumption 4, due to the existence of capacity constraint in mass process, it is impossible to increase new inserted order's volume infinitely. Here we set the new inserted order's volume as not more than *k* times of upper limit of FLSP's normal capacity in mass process. Please see the following formula:

(15) $$\begin{array}{c} N_{J_{0}+1}\leq k\bar{N}. \end{array}$$

Besides, in actual scheduling process, a FLSP's compressed time cannot be longer than the normal operation time itself; namely, *T*_1_^ext^ + *T*_1_ > 0, *T*_2*j*_^ext^ + *T*_2*j*_ > 0 should be fulfilled.

Based on the optimization objectives and constraints above, the whole model established in this paper is as follows:

(16) Min⁡Z1=1∑j=1J0+1Nj(f1+f2+f3)Max⁡Z2=S1+∑j=1J0+1S2j1+J0+1  =Stime,1Squantity,1+∑j=1J0+1S2j1+J0+1subject  toT1+T1ext+T2j+T2jext  +[∑j=1J0+1Nj−N¯]+N¯λT1≤Tjexp⁡(1+βj)Δpro=Nj+1F−[(f1+f2+f3)−(C1T1∑j=1J0Nj+∑j=1J0(C2jT2jNj))]>0S1≥S1L,  S2j≥S2jL,NJ0+1≤k∑j=1J0Nj, T1ext+T1>0T2jext+T2j>0,  j=1,2,…,J0+1.

## 5. Model Solution

### 5.1. Simplifying the Multiobjective Programming Model

The LSSC order insertion scheduling model has two objectives and seven constraints. It is a typical multiobjective programming problem. In this paper, the typical linear weighting method is chosen to solve our model. Objective *Z*_1_ should dimensionally be transformed into a number in the range of [0, 1]. After the mathematical transformation, the synthesized objective function is shown as follows:

(17) $$\begin{array}{c} \max Z=w_{1}\frac{Z_{1}^{\min}}{Z_{1}}+w_{2}Z_{2}, \end{array}$$

where *w*_1_, *w*_2_ represent the weights of *Z*_1_ and *Z*_2_, respectively. *w*_1_ ≥ 0, *w*_2_ ≥ 0, and *w*_1_ + *w*_2_ = 1. *Z*_1_^min⁡^ is the minimum of *Z*_1_ when not considering other objective functions. *Z* is also called the comprehensive performance objective of LSSC.

### 5.2. Using the Genetic Algorithm to Solve the Model

The genetic algorithm is an effective method used to search for the optimal solution by simulating the natural selection process. As it uses multiple starting points to begin the search, it has a satisfactory global search capacity. For the combinatorial optimization problem, the genetic algorithm is quite effective to the solve NP problem, such as the production scheduling problem [31], travelling salesman problem [32], knapsack problem [33], and bin packing problem.

In this paper, instead of comparing or selecting a best method among different kinds of solution methods, we just choose an appropriate method. Given the superiority of the genetic algorithm in solving programming problems and the successful application to scheduling problems [31], this paper uses the genetic algorithm to solve the proposed model.

## 6. Numerical Analysis

By conducting a numerical analysis, this section illustrates the validity of model, explores the influence of relevant parameters on the scheduling results and further gives some effective recommendations for supply chain scheduling and optimization.  Section 6.1 presents the basic data of the numerical example.  Section 6.2 shows the scheduling results.  Section 6.3 discusses the influence of the time delay coefficient *β*_*j*_ of order completion on the scheduling results of the LSSC.  Section 6.4 presents the influence of the new inserted order's volume *N*_*j*_0_+1_ on order insertion decision.  Section 6.5 presents the influence of *β*_*j*_ on *N*_*j*_0_+1_^max⁡^.  Section 6.6 shows the influence of $\bar{N}$ on *N*_*j*_0_+1_^max⁡^.

### 6.1. Numerical Example Description and Basic Data

The parameter values used in our model are shown in Tables 2 and 3.

### 6.2. Numerical Example Results

Genetic algorithm is adopted to solve the problem. It is assumed that the genetic population should be 800 and the hereditary algebra should be 800. And the program for our model is written within MATLAB 7.8 software and run on a PC with 1.6 GHz quad-core processor and 4 GB memories. Computer system is windows 7.0. Let *w*_1_ = *w*_2_ = 0.5 and based on the data in Tables 2 and 3, the calculation result is as follows.

The optimal solution is *Z* = 0.9627 and the corresponding scheduling results are as follows.

Mass service operation stage: *T*_1_^ext^ = −3.0044.

Customized operation stage:

(18) [T21extT22extT23extT24ext] =[−0.00430.00120.0018−3.9113].

According to the calculation results above, it is found that operation time in mass process needs to be compressed when order inserted, and the compressed time is 3.0044 units. Among customized processes of these four orders, the first customer order and the fourth customer order need to be operated in time compressed status, and the second and third customer order need to be delayed a little.

### 6.3. Effects of *β*_*j*_ on the Scheduling Performance of the LSSC

Generally speaking, customers' requirement for a service order's completion time may change, and time compression and delay requirement are both possible, which demands a certain degree of time flexibility in scheduling from the LSI. In model building, *β*_*j*_ < 0 means that the service order needs to be finished ahead of time; accordingly, *β*_*j*_ > 0 means that the service time needs to be delayed. In this section, the influence of the delay (or compression) coefficient of order completion time *β*_*j*_ on *Z* is discussed. With other model parameters unchanged, the results of *Z* are calculated corresponding to the changing *β*_*j*_. For the convenience of calculation, let all *β*_*j*_ be the same value; namely, the time delay coefficient of order completion *β* of all the customer orders are the same. The results are shown in Table 4.

**Figure 4:**
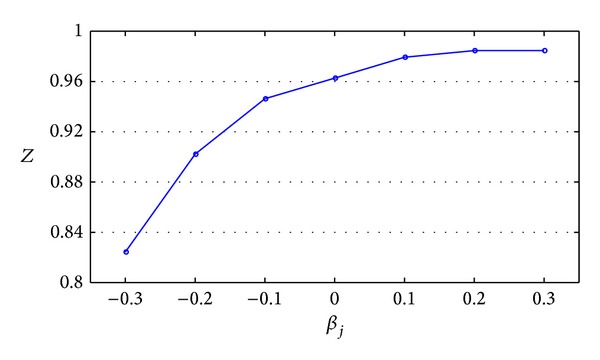
Curve of *Z* changed with *β*_*j*_.

With the data in Table 4 plotted, Figure 4 is obtained.

Based on Table 4 and Figure 4, the following conclusions could be obtained.

(1) With the increase of *β*_*j*_ (from negative to positive), *Z* first increases and then tends to be stable, which means that a reasonable positive tolerance coefficient contributes to achieving the maximal value of comprehensive performance (i.e., in this numerical example, when *β*_*j*_ = 0.2, comprehensive performance reaches the maximum *Z* = 0.9846). Conversely, if *β*_*j*_ is negative, the maximal value of comprehensive performance cannot be reached. Moreover, a smaller time delay tolerance coefficient (i.e., the service should be operated in time compression) results in poorer comprehensive performance. Therefore, it could be inferred that comprehensive performance may deteriorate when customers request shortening the order completion time of FLSP.

(2) If *β*_*j*_ < −0.3, the model has no solution, which means that the LSSC cannot operate in time compression without limit. Furthermore, the LSSC scheduling has certain restriction, and the order cannot be completed as early as the customer wants it.

(3) After *β*_*j*_ reaches a certain level (in this example, it is *β*_*j*_ > 0.2), *Z* tends to be stable. It has no contribution to improve the total performance of supply chain if LSSC continues to increase *β*_*j*_. Therefore, in practice, it makes no sense to blindly negotiate with customer to reach the biggest value of *β*_*j*_.

### 6.4. Effects of *N*_*j*_0_+1_ on the Order Insertion Decision

It is easy to understand that the order insertion decision is affected by the volume of new inserted order, which is denoted by *N*_*j*_0_+1_. In Section 6.4, the effect of *N*_*j*_0_+1_ on the order insertion decision is discussed in detail. Keep other parameters unchanged, just change new inserted order's volume *N*_*j*_0_+1_ and try to find solution to our model. If solution exists, then calculate the corresponding value of *Z* and go on to increase *N*_*j*_0_+1_ until model has no solution. The calculation result is shown in Table 5.

**Figure 5:**
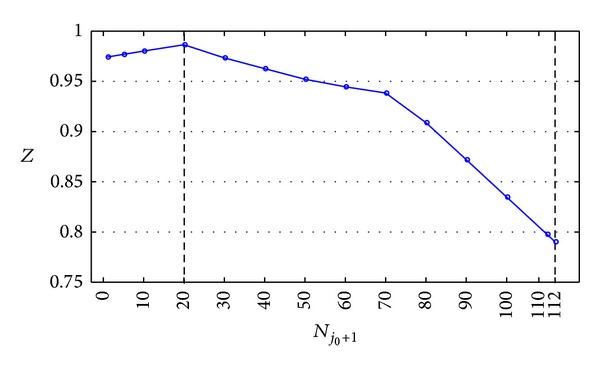
Curve of *Z* varied with *N*_*j*_0_+1_.

With the data in Table 5 plotted, Figure 5 is obtained.

Figure 5 and Table 5 indicate the following conclusions.

(1) Along with the increase of *N*_*j*_0_+1_, comprehensive performance of LSSC *Z* first increases and then tends to be stable. The inflection point of the curve occurs at the point whose value is the difference between normal operation capacity of mass process and the volume of original orders, which is called capacity surplus of mass process in this paper. Therefore, supply chain performance reaches the optimal when the new inserted order's volume is equal to the capacity surplus of mass process. It is easy to understand that in the situation above, new inserted order can be operated together with original orders without increase of extra order preparation time. Moreover, FLSP's normal operation capacity, such as the maximum loading capacity of truck or the maximum capacity of warehouse, is fully utilized in this situation. Hence, FLSP's satisfaction degree of service time and service quantity are both in high level. In this numerical example, it is reflected by the maximum value of *Z*_2_ = 0.9732.

(2) Along with the continuous increase of *N*_*j*_0_+1_ (here *N*_*j*_0_+1_ > 20), comprehensive performance of LSSC *Z* decreases due to two reasons. On the one hand, with the increase of *N*_*j*_0_+1_, original orders' preparation time of mass process increases and resulted in time compression operation in each process. Apparently, extra cost will increase accordingly as well as *Z*_1_. On the other hand, FLSP's satisfaction degree *Z*_2_ will be decreased when their operating order volume exceeds the original schedule. Considering these two factors together, comprehensive performance of LSSC *Z* decreases gradually.

(3) Along with the continuous increase of *N*_*j*_0_+1_ (which is more than 113 units in our example), supply chain cannot operate anymore. In other words, new order cannot be inserted.

### 6.5. Effects of *β*_*j*_ on the Upper Limit of New Order's Insertable Volume *N*_*j*_0_+1_^max^

In our numerical example, there are three original orders and one new inserted order. Thus, *N*_*j*_0_+1_^max⁡^  can be replaced by *N*_4_^max⁡^. Keep other parameters unchanged and change the value of *β*_*j*_. In each value of *β*_*j*_, only change the value of *N*_*j*+1_ and calculate the upper limit of new order's insertable volume *N*_4_ (which is denoted as *N*_4_^max⁡^). As described above, set the benchmark value *β*_*j*_ = [0 0 0 0] and calculate the corresponding *N*_4_^max⁡^ when *β*_*j*_ is taken different values. Then the results are shown in Table 6.

**Figure 6:**
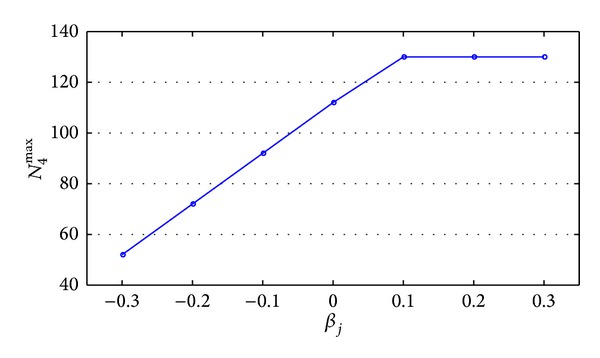
Curve of *N*_4_^max⁡^ varied with *β*_*j*_.

With the data in Table 6 plotted, Figure 6 is obtained.

According to Figure 6, it is found that *β*_*j*_ has significant influence on new order's insertable volume *N*_*j*_0_+1_^max⁡^. From the view of the overall trend, along with the increase of *β*_*j*_, new order's insertable volume *N*_4_^max⁡^ increases and tends to be stable.

### 6.6. Effects of $\bar{N}$ on *N*_*j*_0_+1_^max^

In this section, the effect of upper limit of normal operation capacity of mass process $\bar{N}$ on *N*_*j*_0_+1_^max⁡^ is explored. Keep other parameters unchanged and set ${\bar{N}}_{0}=110$ as benchmark. And *R* is used to be denoted as adjustment coefficient of normal operation capacity of mass process. In calculation, $\bar{N}$ can be presented as $\bar{N}={\bar{N}}_{0}\times \left(1+R\right)$, *R* ∈ (−1, +*∞*). Then change the value of *R* and calculate corresponding upper limit of *N*_*j*+1_, which is denoted by *N*_*j*+1_^max⁡^. This upper limit is the upper limit of insertable volume. Results are shown in Table 7. Basic data $\bar{N}={\bar{N}}_{0}=110$ and corresponding *N*_4_^max⁡^ = 112, which is benchmark value.

**Figure 7:**
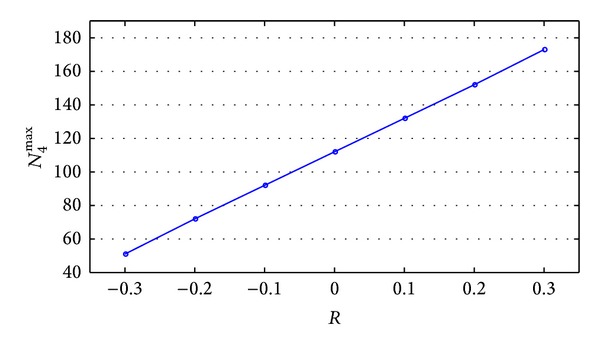
Curve of *N*_4_^max⁡^ varied with *R*.

With the data in Table 7 plotted, Figure 7 could be obtained.

According to Figure 7, the following conclusions could be made.

(1)  $\bar{N}$ has significant influence on new order's insertable volume *N*_*j*_0_+1_^max⁡^. From the view of the overall trend, new order's insertable volume *N*_*j*_0_+1_^max⁡^ increases along with the increase of $\bar{N}$.

(2) See from the view of quantitative relation, the increasing (or decreasing) proportion of new order's insertable volume *N*_*j*_0_+1_^max⁡^ is larger than that of normal operation capacity of mass process $\bar{N}$. As shown in Table 7, in this example, if the adjustment coefficient increases (or decreases) 0.1 time based on the benchmark, namely, increasing (or decreasing) 112 × 0.1 = 11.2 units, absolute value of the increase (or decrease) in *N*_4_^max⁡^ is approximately 20 units, compared to benchmark value. The latter number can be calculated by the subtraction between the former item and the latter item in second column of Table 7. Therefore, intuitively, if $\bar{N}$ increases per unit, the *N*_*j*_0_+1_^max⁡^ increases more than one unit. In this example, the number is approximately 20 ÷ 11.2 = 1.79 units. In consequence, it can contribute to inserting relatively more extra orders for customers to choose a supply chain whose upper limit of normal operation capacity of mass process $\bar{N}$ is relatively large. Furthermore, for LSI, increasing $\bar{N}$ significantly significantly contributes to improving its order insertion capacity.

(3) Supply chain comprehensive performance *Z* is rarely affected by *R* and remains stable. It is found that when operating in capacity limiting conditions (namely, when the new inserted order's volume is the upper limit that supply chain can support), supply chain performance is almost the same. No matter if the upper limit of normal operation capacity of mass process is large or small, there is not big difference in overall performance. This conclusion is opposite to what we guessed and thus very interesting. Generally, it is usually guessed that a supply chain with larger operation capacity in mass process has greater performance when operating in capacity limit conditions. Obviously, any supply chain will show a relatively bad performance when operating in capacity limit conditions, since unit cost is high and FLSP's satisfaction degree is low.

(4) Combined with conclusions in Section 6.5, it is found that both $\bar{N}$ and *β*_*j*_ will significantly influence the maximum volume of insertable order. By comparison, improving the normal operation capacity of mass service process $\bar{N}$ is more useful in increasing maximum volume of insertable order. The reason is that after reaching a certain level (in our example it is 0.1 time of normal completion time), continuous increase in *β*_*j*_ makes no contribution in increasing insertable order volume. However, even if $\bar{N}$ increases to 0.3 time of benchmark, it still makes contribution to increasing maximum insertable order volume. This conclusion is relatively useful for LSI.

## 7. Main Conclusions and Management Insights

This section summarizes main conclusions and further explains related insights for researchers. And management insights for LSI are also discussed, which offers useful recommendations for scheduling decisions.

### 7.1. Main Conclusions Derived from the Scheduling Model

The following conclusions are based on the previous analysis.

(1) On the one hand, the smaller the time delay coefficient *β*_*j*_ of order completion is, the worse the supply chain performance will be. When *β*_*j*_ is less than a certain value, this scheduling model has no solution, which indicates operation time could not be compressed infinitely. On the other hand, if customers permit completion time delay, increase in *β*_*j*_ could improve supply chain comprehensive performance. However, supply chain performance will stop improving but remain stable after increasing to a certain level. Thus, it makes no sense to negotiate with customer blindly for the biggest value of *β*_*j*_ in practice.

(2) The delay coefficient of order completion time *β*_*j*_ permitted by customer obviously influences insertable order volume. Generally, along with the increase of *β*_*j*_, order insertable volume gradually increases and tends to be stable after reaching a certain level.

(3) With inserted order's volume increasing, the comprehensive performance of LSSC *Z* first increases and then decreases. The curve of *Z* inflects at the point representing the difference between normal operation capacity of mass process and the volume of original orders, which is called capacity surplus of mass process in this paper. Therefore, supply chain performs best when the new inserted order's volume is equal to the capacity surplus of mass process. With *N*_*j*_0_+1_ continuously increasing, the comprehensive performance of LSSC *Z* decreases. Furthermore, supply chain cannot operate anymore after new inserted order's volume reaches a certain level.

(4) The upper limit of normal operation capacity $\bar{N}$ has significant influence on new order's insertable volume *N*_*j*_0_+1_^max⁡^. Generally, along with the increase of $\bar{N}$, new order's insertable volume *N*_*j*_0_+1_^max⁡^ increases. Seen from the view of quantitative relation, the increasing (or decreasing) proportion of new order's insertable volume *N*_*j*_0_+1_^max⁡^ is larger than that of normal operation capacity of mass process $\bar{N}$; that is *N*_*j*_0_+1_^max⁡^ increases more than one unit when $\bar{N}$ increases one unit. Therefore, it is useful for customers to choose a supply chain whose has large normal operation capacity of mass process when it is expected to insert relatively more extra orders. Furthermore, it is quite effective to increase $\bar{N}$ when LSI plans to improve its capacity in order insertion.

(5) Both $\bar{N}$ and *β*_*j*_ will significantly influence the maximum volume of insertable order. With comparison, improving the normal operation capacity of mass service process $\bar{N}$ is more useful in increasing maximum volume of insertable order.

### 7.2. Implications for Researchers

This study establishes the LSSC order insertion model considering capacity and time factors and analyzes the order insertion problem in the MCLS environment, which could be referred to by other researchers. First, this study provides theoretical basis for further studies on the scheduling methods and performance optimization methods of LSSCs in the MCLS environment. For example, it is found that both the order completion time delay coefficient permitted by customer and the volume of new inserted order have influence on supply chain comprehensive performance and will further affect the order insertion decisions. Although both the normal operation capacity of mass process and the delay coefficient of order completion time permitted by customer will significantly influence the maximum volume of insertable order, improving the former one is more useful in increasing the maximum volume of insertable order. These conclusions could be useful for further studies on order insertion scheduling models. Second, the order similarity coefficient proposed by this paper provides reference for other researches on supply chain order insertion model. Third, researchers could develop integrated study on order insertion decision and CODP based on our model, and empirical research on that issue could also be conducted. In short, this study could offer a basic theoretical foundation for further studies on LSSC scheduling.

### 7.3. Implications for Managers

This research is developed on the background of MCLS, and the conclusions presented in this paper could serve as reference for the participants in LSSC, especially LSI. Specifically, three important points are shown as follows.

1. For customers, it is useful to choose a supply chain whose normal operation capacity of mass process is relatively large for inserting relatively more extra orders. Thus, LSI should make efforts to improve their service capacity in mass process to face the challenge from newly increased order's demand.
2. Supply chain performance reaches the optimal when the new inserted order's volume is equal to the capacity surplus of mass process. Besides, when a certain level is achieved, new order cannot be inserted and supply chain operation breaks down. Hence, it is sensible for LSI to choose the new order whose volume matches the capacity surplus of mass process to reach optimal supply chain performance.
3. The insertable volume of new inserted order is affected by both order completion time requirements from customers and FLSP's normal operation capacity in mass process. And to increase service capacity in mass process is more useful for improving order insertion capacity. Therefore, LSI had better enhance the operation capacity instead of asking customer's permission for delaying completion time.

### 7.4. Research Limitations and Directions for Future Research

With full consideration of service capacity and time factor, an order insertion scheduling model of LSSC is established, aiming to minimize the average unit volume operation cost of the LSI and maximize the average satisfaction degree of FLSPs. And in order to verify the viability and effectiveness of our model, a specific example is numerically analyzed with MATLAB 7.8 software. Furthermore, effects that the order completion time delay coefficient permitted by customer and the new inserted order's volume have on supply chain comprehensive performance are discussed, as well as effects that the new inserted order's volume and the upper limit of normal operation capacity in mass process have on order insertion decisions. Many useful conclusions are obtained to improve LSI's time scheduling decision. However, this paper has several limitations. For example, the model solution and analysis are obtained with a numerical example, which may not represent all situations in reality. Besides, the influences of order insertion scheduling on CODP is not considered in our model. In practice, insertion of new order may cause CODP changing, which could be researched in future work. What is more, in our model, we assume that there is only one new arrived order that needs to be inserted and do not consider multiorder insertion problem. In the future, the multiorder insertion problem could be explored based on the order priority.

## Figures and Tables

**Table 1:** Notations for the model.

Notations	Description
*C*_1_	The normal service cost per unit time per unit quantity of the FLSP in mass process in offering mass operation; this cost is the normal cost without time compression or delay in the operation when operated according to original schedule

*C*_1_^ext^	The extra service cost per unit time per unit quantity of the FLSP in mass process in offering mass operation; this cost is the extra cost due to inserting new order and rescheduling which caused extra time compression or delay in completion orders

*C*_2*j*_	The normal service cost per unit time per unit quantity in offering customized operation for the *j*th customer order; this cost is the normal cost without time compression or delay in the operation when operated according to original schedule

*C*_2*j*_^ext^	The extra service cost per unit time per unit quantity of the FLSP in offering customized operation for the *j*th customer order; this cost is the extra cost due to inserting new order and rescheduling which caused extra time compression or delay in completion orders

*C*_*j*_^delay^	The penalty cost per unit time per unit quantity of the *j*th customer order as the order completion time is delayed; *j* = 1,2,…, *J*_0_, *J*_0_ + 1

*F*	The new order's price for per unit time per unit quantity offered by new order's customer

*k*	Since mass process has capacity limit, new order volume cannot increase infinitely; the new order volume is set to be no more than *k* times of the FLSP's normal upper limit capacity in mass service stage; *k* > 0

$\overset{-}{N}$	The upper limit of FLSP's capacity in mass process, which is the upper limit of FLSP's capacity after scheduling according to original orders (due to real limitations, this upper limit may just be the sum volume of original orders or may be larger than the sum volume; if FLSP operates within this limit volume, operation time will not increase)

*N*_*j*_	Volume of the *j*th order; *j* = 1,2,…, *J*_0_, *J*_0_ + 1

*N*_*j*_0_+1_	Volume of the new inserted order, where subscript *J*_0_ + 1 characterizes the new inserted order, the same below

*N*_*j*_0_+1_^max⁡^	The maximum of new inserted order's volume; in our numerical example, there are three original orders and one new inserted order; thus, *N*_*j*+1_^max⁡^ can be replaced by *N*_4_^max⁡^

*S*_quantity,1_	Service quantity satisfaction (capacity) degree of the mass service provider

*S*_time,*i*_	The *i*th FLSP's service time satisfaction degree, *i* = 1,2*j*; *j* = 1,2,…, *J*_0_ + 1

*S*_quantity,1_^0^	Initial value of service quantity (capacity) satisfaction degree of the mass service provider

*S*_1_	The satisfaction degree of the mass process provider

*S*_1_^*L*^	The lower limit of the satisfaction degree of the mass process provider

*S*_2*j*_	The satisfaction degree of the customized process of the *j*th customer order; *j* = 1,2,…, *J*_0_ + 1

*S*_2*j*_^*L*^	The lower limit of the satisfaction degree of the customized process of the *j*th customer order; *j* = 1,2,…, *J*_0_ + 1

*S*_*i*_	Satisfaction degree of the *i*th provider, *i* = 1,2; *j* = 1,2,…, *J*_0_ + 1

*T*_1_	The normal operation time of original orders before new order's arrival in mass process; this normal operation time is generated by original order's scheduling result and is input parameter in numerical analysis

*T*_2*j*_	The normal operation time of the *j*th original order before new order arrival in customized process. *j* = 1,2, 3,…, *J*_0_, the same below

*T*_*j*_^exp⁡^	Completion time requirement of the *j*th customer order asked by customers; *j* = 1,2,…, J_0_, *J*_0_ + 1

*T*_*j*_	Actual completion time of the *j*th customer order; *j* = 1,2,…, J_0_, *J*_0_ + 1

*T*_1_^ext^	Extra operation time of the provider in mass service process

*T*_2*j*_^ext^	Extra operation time of the *j*th customer order in customized process

*w*_1_	The weight of objective function *Z*_1_ in *Z*

*w*_2_	The weight of objective function *Z*_2_ in *Z*

*Z*_1_	The total cost of LSI

*Z*_2_	The average satisfaction of all processes in LSSC

*Z*_1_^min⁡^	The minimum of *Z*_1_ when not considering the objective functions *Z*_2_

*Z*	The objective function synthesized by *Z*_1_ and *Z*_2_, which is also called the comprehensive performance of LSSC

Z*	The optimal value of *Z*

*λ*	Similarity coefficient of new inserted order and original orders

*λ*_1_	Time requirement similarity coefficient of new inserted order and original orders

*λ*_2_	Service procedure similarity coefficient of new inserted order and original orders

*λ*_3_	Customer service product similarity coefficient of new inserted order and original orders

*β*_*j*_	The delay coefficient of the order completion time permitted by the *j*th customer for its order

Δpro	Profit increase resulted by new inserted order

Note: *T*_1_^ext^, *T*_2*j*_^ext^ are decision variables.

**Table 2:** Basic data (1).

Parameter	*T*_1_	*C*_1_	*C*_1_^ext^	$\bar{N}$	*S*_quantity,1_^0^	*S*_quantity,1_^*L*^	*F*	*λ*_2_	*λ*_3_	*k*
Value	29	10	18	110	0.3	0.5	3000	0.6	0.7	3

**Table 3:** Basic data (2).

Parameter	*j* = 1	*j* = 2	*j* = 3	*j* = 4 (new inserted order)
*T*_2*j*_	24	31	36.5	30
*T*_*j*_^exp⁡^	53	60	65.5	55
*C*_*j*_^delay^	19	22	17	20
*N*_*j*_	20	30	40	40
*β*_*j*_	0	0	0	0
*C*_2*j*_	28	22	37	32
*C*_2*j*_^ext^	35	41	44	35

**Table 4:** The influence of *β*_*j*_ on comprehensive performance of LSSC *Z*.

*β*_*j*_	*Z*
$\left[\begin{array}{cccc} -0.4 & -0.4 & -0.4 & -0.4 \end{array}\right]$	No solution
$\left[\begin{array}{cccc} -0.3 & -0.3 & -0.3 & -0.3 \end{array}\right]$	0.8243
$\left[\begin{array}{cccc} -0.2 & -0.2 & -0.2 & -0.2 \end{array}\right]$	0.9022
$\left[\begin{array}{cccc} -0.1 & -0.1 & -0.1 & -0.1 \end{array}\right]$	0.9463
$\left[\begin{array}{cccc} 0 & 0 & 0 & 0 \end{array}\right]$	**0.9627**
$\left[\begin{array}{cccc} 0.1 & 0.1 & 0.1 & 0.1 \end{array}\right]$	0.9793
$\left[\begin{array}{cccc} 0.2 & 0.2 & 0.2 & 0.2 \end{array}\right]$	0.9846
$\left[\begin{array}{cccc} 0.3 & 0.3 & 0.3 & 0.3 \end{array}\right]$	0.9846

**Table 5:** Effect of *N*_*j*_0_+1_ on comprehensive performance of LSSC *Z*.

*N*_*j*_0_+1_	*Max*⁡*Z*	*Z*_1_	*Z*_2_
1	0.9743	1.2690*e* + 003	0.9491
5	0.9769	1.2737*e* + 003	0.9540
10	0.9802	1.2795*e* + 003	0.9605
20	0.9865	1.2896*e* + 003	0.9732
30	0.9734	1.3247*e* + 003	0.9472
40	0.9627	1.3580*e* + 003	0.9256
50	0.9521	1.3940*e* + 003	0.9079
60	0.9446	1.4253*e* + 003	0.8930
70	0.9384	1.4516*e* + 003	0.8777
80	0.9089	1.5218*e* + 003	0.8180
90	0.8720	1.6059*e* + 003	0.7440
100	0.8348	1.6890*e* + 003	0.6698
110	0.7977	1.7709*e* + 003	0.5955
112	0.7902	1.7874*e* + 003	0.5805
113	No solution		

**Table 6:** Effect of *β*_*j*_ on *N*_*j*_0_+1_^max⁡^.

*β*_*j*_	*N*_4_^max⁡^	Compared with benchmark value, the growth proportion of *N*_4_^max⁡^
$\left[\begin{array}{cccc} -0.3 & -0.3 & -0.3 & -0.3 \end{array}\right]$	52	−53.6%
$\left[\begin{array}{cccc} -0.2 & -0.2 & -0.2 & -0.2 \end{array}\right]$	72	−35.7%
$\left[\begin{array}{cccc} -0.1 & -0.1 & -0.1 & -0.1 \end{array}\right]$	92	−17.9%
**Benchmark ** $\left[\begin{array}{cccc} 0 & 0 & 0 & 0 \end{array}\right]$	**112**	**—**
$\left[\begin{array}{cccc} 0.1 & 0.1 & 0.1 & 0.1 \end{array}\right]$	130	16.1%
$\left[\begin{array}{cccc} 0.2 & 0.2 & 0.2 & 0.2 \end{array}\right]$	130	16.1%
$\left[\begin{array}{cccc} 0.3 & 0.3 & 0.3 & 0.3 \end{array}\right]$	130	16.1%

**Table 7:** Effect of $\bar{N}$ on *N*_*j*_0_+1_^max⁡^.

Adjustment coefficient *R*	*N*_4_^max⁡^	Compared with benchmark value, the growth proportion of *N*_4_^max⁡^	*Z*	*Z*_1_	*Z*_2_
−0.3	51	−54.5%	0.7925	1.7713*e* + 003	0.5849
−0.2	72	−35.7%	0.7885	1.7849*e* + 003	0.5770
−0.1	92	−17.9%	0.7896	1.7861*e* + 003	0.5791
**0** (benchmark)	**112**	**—**	**0.7902**	1.7874**e** + 003	**0.5805**
0.1	132	17.9%	0.7910	1.7881*e* + 003	0.5821
0.2	152	35.8%	0.7916	1.7889*e* + 003	0.5831
0.3	173	54.5%	0.7892	1.7956*e* + 003	0.5784
